# Bioequivalent Letermovir Usage for Prophylaxis in Haploidentical Stem Cell Transplantation at High Risk of CMV Reactivation: A Report of Two Cases From India

**DOI:** 10.7759/cureus.83035

**Published:** 2025-04-26

**Authors:** Arijit Nag, Baalamurugan KT, Jeevan Kumar, Shouriyo Ghosh, Dibakar Podder, Debranjani Chattopadhyay, Saswata Saha, Rizwan Javed, Soumyadip Chatterji, Asish Rath, Reena Nair, Suyash Bharat

**Affiliations:** 1 Department of Clinical Hematology and Cellular Therapies, Tata Medical Center, Kolkata, IND; 2 Department of Infectious Diseases, Tata Medical Center, Kolkata, IND; 3 Department of Lab Hematology, Tata Medical Center, Kolkata, IND; 4 Department of Medical Affair, Zydus Lifesciences Ltd, Ahmedabad, IND

**Keywords:** classical hodgkin lymphoma, cmv prophylaxis, haploidentical sct, letermovir, wiskott-aldrich syndrome

## Abstract

Cytomegalovirus (CMV) reactivation is a major complication in allogeneic hematopoietic stem cell transplantation (HSCT), increasing nonrelapse mortality (NRM) and transplant-related complications. Letermovir, a CMV deoxyribonucleic acid (DNA) terminase inhibitor, has demonstrated efficacy in reducing CMV reactivation without the hematologic toxicity associated with traditional antivirals.

We report two HSCT cases, one with a T-cell receptor (TCR) alpha/beta-depleted haploidentical transplant and another haploidentical (6/10 HLA-matched) stem cell transplant, both at significant risk for CMV reactivation. Letermovir prophylaxis was initiated early post-transplant and continued for 100-200 days, depending on risk factors. Both patients remained free of CMV reactivation throughout follow-up (beyond Day +140), with no breakthrough CMV infection or drug-related adverse effects.

This case series highlights the first real-world use of letermovir, India’s bioequivalent letermovir, in haploidentical transplant recipients, supporting its clinical effectiveness in a resource-limited setting. Real-world outcomes were consistent with previously reported clinical trial data. While outcomes were favorable, the small sample size and single-center experience represent limitations. Nonetheless, the findings highlight the potential benefit of extended prophylaxis beyond Day 100 and the need for individualized CMV prevention strategies in immunosuppressed populations.

## Introduction

Cytomegalovirus (CMV) is a significant cause of morbidity and mortality in immunocompromised patients, particularly in those undergoing allogeneic hematopoietic stem cell transplantation (HSCT) [1,2]. The incidence of CMV infection within the first year post-HSCT varies from 24.8% to 61.2%, depending on transplant type and immunosuppressive regimen. One study by Chorão et al. reported that 46% of post-allogeneic HSCT patients had at least one episode of CMV reactivation, with the one-year incidence being 39% for matched sibling donors, 44% for matched unrelated donors, and 62% for haploidentical donors [3,4]. In the absence of effective prophylaxis, CMV reactivation can significantly increase nonrelapse mortality (NRM), primarily due to opportunistic infections and multiorgan dysfunction [5]. Traditional CMV management strategies rely on preemptive therapy with valganciclovir, ganciclovir, or foscarnet, which, despite their efficacy, are associated with myelosuppression, nephrotoxicity, and other toxicities, limiting their use in the early post-transplant period when patients are most vulnerable [6,7]. The need for an effective, well-tolerated CMV prophylactic agent has been a long-standing challenge in transplant medicine, particularly due to the toxicity of traditional antivirals, which often limit their use in the early post-transplant period.

Letermovir, a first-in-class CMV deoxyribonucleic acid (DNA)-terminase complex inhibitor, was approved by the U.S. Food and Drug Administration (FDA) and the European Medicines Agency (EMA) in 2017 for primary CMV prophylaxis in adult CMV-seropositive allogeneic HSCT recipients [8]. Unlike traditional antiviral agents, letermovir specifically targets the CMV UL56 subunit, disrupting viral replication without affecting host cell DNA polymerases, thereby minimizing hematologic toxicity [9]. In India, a bioequivalent generic formulation of letermovir (ANVIMO) has recently been approved by the Central Drugs Standard Control Organization (CDSCO) following recommendations from the Subject Expert Committee (SEC) in its 04th/24 meeting. The approval was granted based on a submitted bioequivalence (BE) study report, with a Phase III clinical trial waiver, for the manufacturing and marketing of letermovir tablets 240 mg and 480 mg [10]. Its introduction is expected to improve transplant outcomes by significantly reducing CMV reactivation rates, CMV-related complications, and NRM, particularly in settings where CMV-seropositive donor-recipient mismatches are prevalent.

## Case presentation

Method

**Figure 1:**
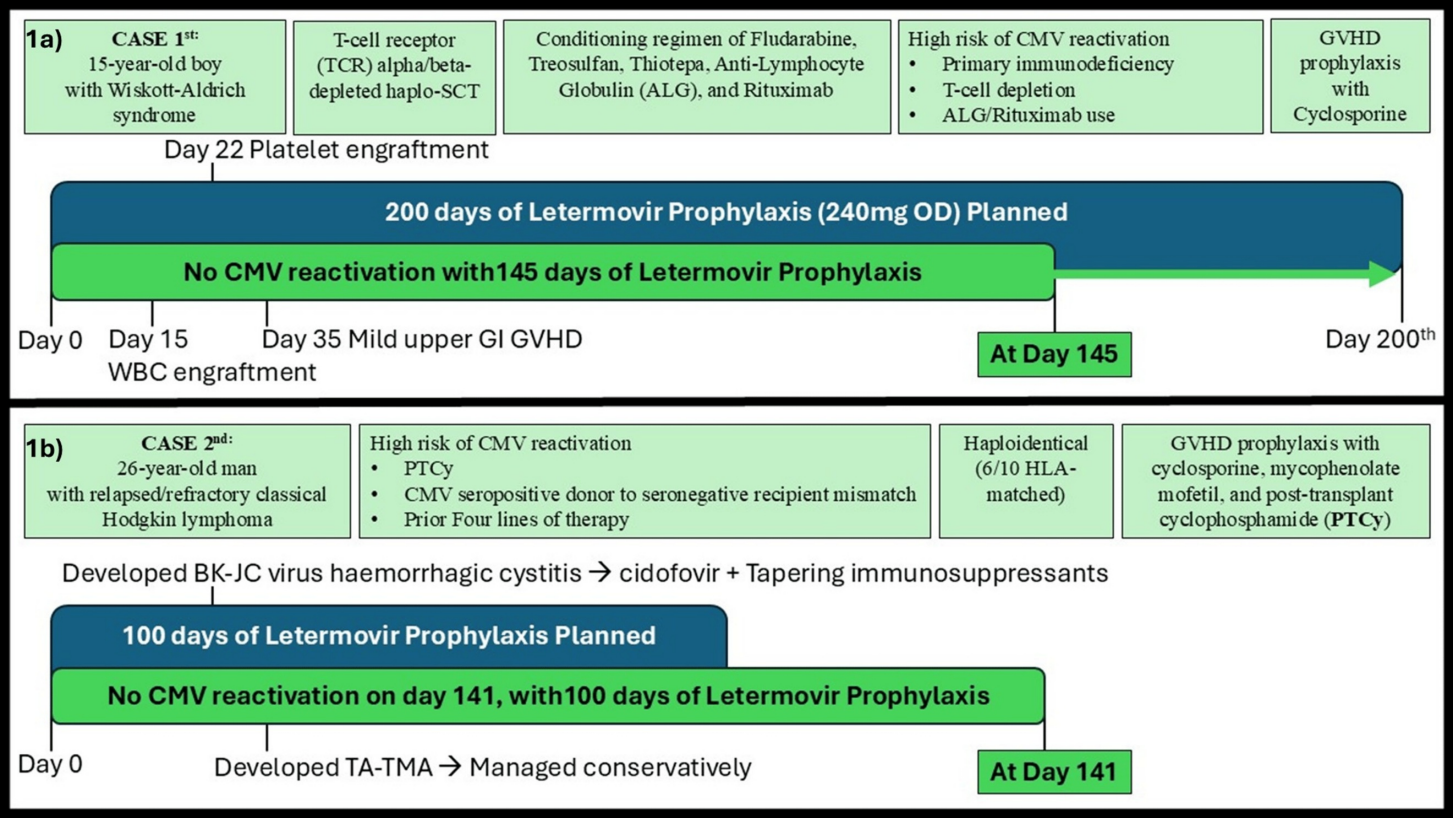
(a) Clinical course of a Wiskott-Aldrich syndrome patient on letermovir prophylaxis post haplo-SCT; (b) clinical course of a classical Hodgkin lymphoma patient on letermovir prophylaxis post haplo-SCT SCT:  stem cell transplantation

This case series includes two patients who underwent haploidentical stem cell transplantation (haplo-SCT) and received letermovir for CMV prophylaxis (Figures 1A, 1B). Both patients were identified as high risk for CMV reactivation based on their underlying conditions, transplant approach, and immunosuppressive regimen. Letermovir (ANVIMO) prophylaxis was initiated on Day 0 post-transplant and continued for 200 days in both patients, based on clinical judgment and in accordance with FDA-approved dosing guidelines (Figure 1a for case 1; Figure 1b for case 2). There were predefined stopping rules based on standard clinical protocols; letermovir was to be discontinued in the event of breakthrough CMV infection, intolerance, or significant drug-related toxicity. Weekly CMV DNAemia monitoring was performed using quantitative polymerase chain reaction (PCR) assays throughout prophylaxis. Adverse events were assessed through routine clinical evaluations and laboratory monitoring. Attribution of adverse events to letermovir was based on clinical judgment, temporal association, and exclusion of other causes common in the post-transplant setting. Known significant adverse events associated with letermovir, such as atrial fibrillation, nausea, diarrhea, vomiting, peripheral edema, cough, headache, fatigue, and abdominal pain, were monitored. Neither patient experienced any grade ≥2 adverse effects attributable to letermovir during the prophylaxis period. The letermovir used in both cases was manufactured and marketed by Zydus Lifesciences.

The conditioning regimen for each patient varied according to disease-specific protocols. One patient received a T-cell replete (TCR) alpha/beta-depleted (TCD) haplo-SCT with CD45RA-depleted addback with a fludarabine, treosulfan, thiotepa, anti-lymphocyte globulin (ALG), and rituximab-based regimen. He was administered cyclosporine monotherapy as graft-versus-host disease (GVHD) prophylaxis. The other patient received fludarabine and treosulfan as a conditioning regimen for a TCR peripheral blood stem cell graft with a standard combination of cyclosporine, mycophenolate mofetil, and post-transplant cyclophosphamide (PTCY). Post-transplant monitoring included engraftment kinetics, GVHD occurrence, viral reactivation screening, and immune reconstitution assessment.

A written informed consent was obtained from both patients or their legal guardians for treatment and data publication. Confidentiality was maintained, and no identifying information has been disclosed in this report.

Case Description

Case 1: A 15-year-old boy was diagnosed with Wiskott-Aldrich syndrome (WAS) based on clinical phenotype of recurrent bleeding, hematological findings of thrombocytopenia with microplatelets, and confirmatory genetic testing. A hemizygous missense mutation (c.397 G>A, p.Glu133Lys) in exon 3+4 of the WAS gene was identified. Initial management included supportive care, intermittent antimicrobial therapy, and platelet transfusions. However, with increasing bleeding episodes and infectious complications during adolescence, haplo-SCT from his father was planned due to the unavailability of a matched unrelated donor.

The transplant involved a T-cell receptor (TCR) alpha/beta-depleted haplo-SCT, using a conditioning regimen of fludarabine, treosulfan, thiotepa, antilymphocyte globulin (ALG), and rituximab. A CD34+ cell dose of 15 × 10⁶ cells/kg was infused, with CD3+ T-cell addback (1 million/kg) on Day +1. GVHD prophylaxis was provided with cyclosporine, targeting a trough level of 100-150 ng/mL. Given the high risk of CMV reactivation due to primary immunodeficiency, T-cell depletion, and ALG/rituximab use, letermovir prophylaxis was initiated at an age- and weight-appropriate dose of 240 mg once daily, considering concurrent cyclosporine use with a planned duration until 200 days. The drug was provided under a named-patient compassionate use program, at the request of the transplant physician and in accordance with FDA and the Drugs Controller General of India (DCGI) prescribing information. White blood cell engraftment occurred on Day +13, and platelet engraftment occurred by Day +22. Mild upper gastrointestinal GVHD developed on Day +37 but was effectively managed with budesonide capsules. Post-transplant infections, including CMV, Epstein-Barr virus, human herpesvirus-6, and adenovirus, were closely monitored. Prophylactic intravenous immunoglobulin (IVIG) was administered biweekly for two months, then monthly until B-cell recovery. No CMV reactivation has occurred during letermovir prophylaxis. By Day +145, the patient had no CMV reactivation while on letermovir prophylaxis and experienced no significant adverse effects.

Case 2: A 28-year-old man was diagnosed with relapsed/refractory classical Hodgkin lymphoma (cHL). The treatment history included four lines of therapy: first-line (adriamycin, bleomycin, vinblastine, dacarbazine (ABVD)), second-line escalated (bleomycin, etoposide, doxorubicin, higher doses of cyclophosphamide, vincristine, procarbazine, and prednisone (ABVD)), followed by third-line bendamustine with brentuximab, and fourth-line nivolumab combined with ifosfamide, carboplatin, etoposide (ICE). At the end of the fourth-line salvage chemo-immunotherapy, he achieved good partial remission but remained refractory. Given the high risk of disease recurrence, he was planned for consolidation with an allogeneic HSCT (allo-HSCT).

The haploidentical (6/10 HLA-matched) SCT was performed on 23/09/2024. The conditioning regimen consisted of fludarabine and treosulfan (10 mg/m²), and a CD34+ cell dose of 4.38 million cells/kg was given. GVHD prophylaxis was provided with cyclosporine, mycophenolate mofetil, and PTCY (50 mg/kg on Days +3 and +4). Considering the high risk for CMV reactivation due to the use of PTCY and a CMV-seropositive donor to seronegative recipient mismatch, letermovir prophylaxis was initiated at an age and weight-appropriate dose of 240 mg once daily from Day 0, with planned administration until Day +100 through a compassionate access program. The post-transplant period was complicated by BK-JC virus hemorrhagic cystitis, which was managed with cidofovir, supportive care, and gradual tapering of immunosuppressants. Additionally, transplant-associated thrombotic microangiopathy (TA-TMA) was developed, which was managed conservatively. At Day 141 post-transplant, the patient remains clinically stable with no evidence of CMV reactivation.

## Discussion

The introduction of letermovir as a prophylactic agent has altered the CMV management landscape, offering a targeted and well-tolerated approach to reducing CMV reactivation without the myelosuppressive and nephrotoxic effects, which are the major hurdle with other antivirals like valganciclovir. The pivotal phase III trial by Marty et al. demonstrated that letermovir prophylaxis in allo-HSCT recipients significantly reduced clinically significant CMV infection at 14 weeks post-transplant (letermovir 19.1% vs. placebo 50.0%, p < 0.001) and at 24 weeks post-transplant (letermovir 18.9% vs. placebo 44.3%, p < 0.001), with a 50% reduction in CMV-related complications and a trend toward improved survival [11]. Importantly, letermovir did not increase the risk of GVHD or other transplant-related complications, supporting its favorable safety profile and clinical effectiveness. Our findings align with these results, as both patients did not experience CMV reactivation despite multiple high-risk factors. A post-hoc analysis of the phase III trial further demonstrated a 42% reduction in one-year NRM among patients who remained CMV-free at Week 14. In our study, both patients remained without CMV reactivation for more than 140 days, supporting the real-world effectiveness of letermovir in preventing both early and late CMV infection in high-risk HSCT recipients.

The optimal duration of letermovir prophylaxis remains an area of active investigation. A study by Liu et al. found that discontinuation of letermovir at Day 100 led to a significant increase in late CMV reactivation and CMV-related mortality, particularly between Days 180 and 365 [12]. The study reported a CMV reactivation rate of 19.5% at Day 180 and 24.8% at Day 365 in the letermovir group, compared to 39.1% and 41.3%, respectively, in those who did not receive prophylaxis.

Similarly, a study by Lin et al. focusing on CMV reactivation patterns in PTCY recipients found that 23% of patients developed clinically significant CMV infection within 90 days of letermovir withdrawal, despite a median prophylaxis duration of 203 days [13]. Additionally, hypogammaglobulinemia (IgG <650 mg/dL) at the time of letermovir discontinuation was strongly associated with a higher risk of late CMV reactivation. This highlights the importance of individualized prophylaxis strategies, particularly in patients receiving PTCY or those with poor immune reconstitution. In our study, Patient 2, who received PTCY, remained CMV-free throughout the prophylaxis period, showcasing letermovir’s efficacy in this high-risk subgroup.

One concern regarding letermovir is its potential effect on CMV-specific T-cell reconstitution, which could increase the risk of late CMV reactivation after prophylaxis discontinuation. However, a study by Marty et al. analyzing patients with detectable CMV DNA at randomization in the phase III trial found that letermovir still significantly reduced progression to CMV disease (64.6% vs. 90.9%), despite some patients already having low-level viremia at baseline. The study used a viral load threshold of >137 IU/mL (151 copies/mL) for defining high-risk patients and >273 IU/mL (300 copies/mL) for low-risk patients with detectable DNAemia [14]. This suggests that letermovir effectively prevents early CMV-related complications, even in patients with some degree of viral replication at transplant.

In pursuit of decreasing late CMV reactivation in high-risk transplant cases, a multicenter, randomized, double-blind, placebo-controlled phase III trial by Russo et al. demonstrated that extending letermovir prophylaxis to 200 days significantly reduced late CMV infection (3% in the 200 days extended letermovir prophylaxis group vs. 19% in the standard 100 days letermovir prophylaxis group; p = 0.0005) [15]. This study reinforces the benefit of prolonged prophylaxis in high-risk CMV reactivation HSCT recipients, supporting the approach taken in our case series, where Patient 1, who underwent TCR alpha/beta-depleted haploidentical transplantation, was planned for extended letermovir prophylaxis up to Day 200 to mitigate late CMV reactivation risk.

While these cases demonstrate favorable real-world outcomes using India’s first bioequivalent generic letermovir (ANVIMO), the findings must be interpreted with caution. The small sample size, absence of a control group, and single-center nature of this series limit generalizability. Furthermore, immune reconstitution kinetics, though clinically monitored, were not comprehensively analyzed or reported, which remains a limitation. Larger, prospective studies are warranted to validate these observations, assess long-term outcomes, and better define optimal prophylaxis duration in diverse transplant settings. Although letermovir has a favorable safety profile and minimal hematologic toxicity, concerns about potential antiviral resistance, particularly with prolonged use, remain relevant and require continued surveillance. Additionally, cost considerations, especially in resource-constrained healthcare environments, may limit the widespread adoption of extended prophylaxis protocols and should be factored into future health economic evaluations.

## Conclusions

This case series highlights the clinical effectiveness of bioequivalent generic letermovir (ANVIMO) prophylaxis in preventing CMV reactivation in high-risk haploidentical HSCT recipients. Both patients remained CMV-free despite multiple risk factors, aligning with previously reported clinical trial outcomes. Extending letermovir prophylaxis beyond Day 100, targeting up to 200 days, may be particularly beneficial in individuals at high risk for late CMV reactivation, supporting the need for individualized prophylaxis strategies. However, the small sample size and single-center nature of this report limit the generalizability of the findings. Larger prospective studies or registry-based audits in Indian cohorts are warranted to validate these observations and to optimize CMV prevention strategies in real-world transplant settings.
